# Effects of pioglitazone on nonalcoholic steatohepatitis in a patient with anorexia nervosa: A case report

**DOI:** 10.3892/etm.2014.1509

**Published:** 2014-01-28

**Authors:** TOMOHIKO OHNO, YOICHI NISHIGAKI, TETSUYA YAMADA, YUKO WAKAHARA, HIROYASU SAKAI, KOTARO YOSHIMURA, MASAHITO SHIMIZU, TOSHIO USUI, MASAYA SAITO, ICHIRO YASUDA, HISASHI TSURUMI, EIICHI TOMITA, HISATAKA MORIWAKI

**Affiliations:** 1 Department of Medicine, Gifu University Graduate School of Medicine, Gifu 501-1194, Japan; 2 Second Department of Internal Medicine, Gifu Municipal Hospital, Gifu 500-8513, Japan; 3 Department of Clinical Laboratory, Gifu Municipal Hospital, Gifu 500-8513, Japan; 4 Department of Internal Medicine, Seki Central Hospital, Gifu 500-3919, Japan

**Keywords:** steatohepatitis, malnutrition, pioglitazone

## Abstract

Diseases associated with metabolic syndromes are of major concern in developed countries. Nonalcoholic steatohepatitis (NASH) is one of the manifestations of metabolic syndrome in the liver. Previous studies have shown that NASH is also caused by malnutrition. In the present study, a case of malnutrition-associated NASH in a 66-year-old female with anorexia nervosa is reported. The patient had a body mass index (BMI) of only 11.1 kg/m^2^ and serum alanine aminotransferase levels of 1,495 IU/l. Steatohepatitis with fibrosis was confirmed by percutaneous liver needle biopsy. Total parenteral nutrition was conducted at first, followed by the administration of Stronger Neo-Minophagen C (a glycyrrhizin-containing preparation), ursodeoxycholic acid and prednisolone. The abnormal elevation of aminotransferase levels of the patient was prolonged and total bilirubin levels increased. Pioglitazone (15 mg/day), which has been identified to be effective for nonalcoholic steatohepatitis, was then administered. This resulted in marked reductions in aminotransferase and bilirubin levels within three months. Histological improvement of the liver was also confirmed by percutaneous liver needle biopsy after one year. The observations in the present case suggest that pioglitazone may be useful for the treatment of malnutrition-associated NASH.

## Introduction

Anorexia nervosa (AN) is a mental disorder that is common worldwide. In 1999, the overall age and gender-adjusted incidence rate was reported to be 8.3 cases per 100,000 person-years, and a long-term linear increase in the incidence rate of AN was observed, particularly in adolescent females (1). Approximately 30% of patients with AN show liver dysfunction (2,3) and histological findings of the liver indicate similar results to those of patients with nonalcoholic steatohepatitis (NASH) (4). NASH is classified into two types: Primary NASH associated with a lifestyle-related disease, such as metabolic syndrome and secondary NASH following other diseases (5). Thus, malnutrition-associated NASH is classified into secondary NASH (6). Generally, liver dysfunction complicated by malnutrition improves immediately after the administration of total parenteral nutrition (TPN) (7–9); however, it occasionally develops into acute hepatic failure (4) and no established therapy is available in such cases.

Primary NASH is an obesity-associated disease and weight reduction therapy is the only remedy that has been confirmed to be effective (10). However, weight reduction therapy has limitations, as a number of patients with NASH often fail to reduce their weight. Furthermore, weight reduction therapy is not indicated for patients with secondary NASH without obesity. Thus, several clinical trials have been conducted to investigate alternative therapies for NASH. Pioglitazone was the first drug whose effectiveness for NASH was confirmed in a randomized control trial (11). Therefore, pioglitazone may also be a potential therapeutic agent for patients with secondary NASH.

The present study reports a case of secondary NASH with AN in a 66-year-old woman, and the effects of pioglitazone on liver function and histological findings are presented. This study was approved by the Ethics Committee of Gifu University Graduated School of Medicine (Gifu, Japan). The patient consented to the publication of this study.

## Case report

A 66-year-old female with AN was referred to the Division of Internal Medicine at Seki Central Hospital (Gifu, Japan) in June 2008 for liver dysfunction. The patient neither consumed alcohol nor was infected with hepatitis. Computed tomography (CT) and ultrasonography (US) showed normal findings of the liver. The patient was extremely thin and had a body mass index (BMI) of only 11.1 kg/m^2^. Needle biopsy of the liver was not performed at this time. The patient continued to restrict dietary intake in spite of counseling in the hospital and her general condition gradually deteriorated. In January 2009 the patient felt severe general fatigue and was not able to eat. Finally, the patient agreed to hospitalization on January 14, 2009 due to severe malnutrition and liver dysfunction.

**Figure 1:**
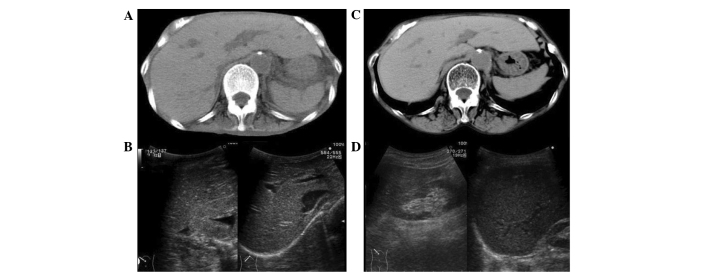
Imaging results of the patient’s liver. (A) Computed tomography and (B) ultrasound findings on admission; and (C) computed tomography and (D) ultrasound findings after 1 year.

On admission, the height and weight of the patient were 150 cm and 25 kg, respectively, corresponding to the same BMI of 11.1 kg/m^2^. The body temperature was 37.4°C, blood pressure was 120/64 mmHg, heart rate was 97 beats/min and O_2_ saturation was 98%. The patient’s eyes were neither anemic nor icteric. Cardiovascular, pulmonary, abdominal and neurological examinations showed no abnormalities, but the skin was observed to be dry with decreased turgor. Serum aspartate aminotransferase (AST) and alanine aminotransferase (ALT) levels were elevated to 3,665 IU/l and 1,495 IU/l, respectively. Serological tests for HBsAg and anti-HCVAb were negative. The prothrombin time was 52%. Elevated levels of serum bilirubin, creatinine and urea nitrogen were also observed. Thyroid hormone levels were normal (Table I). Abdominal CT showed a normal liver and the liver/spleen attenuation ratio was 1.08 (hepatic CT attenuation was 65.6 Hu). Due to severe emaciation, subcutaneous or visceral fat was hardly observed (Fig. 1A). Abdominal US showed a homogeneous liver pattern and the liver/kidney contrast was not enhanced (Fig. 1B).

**Figure 2:**
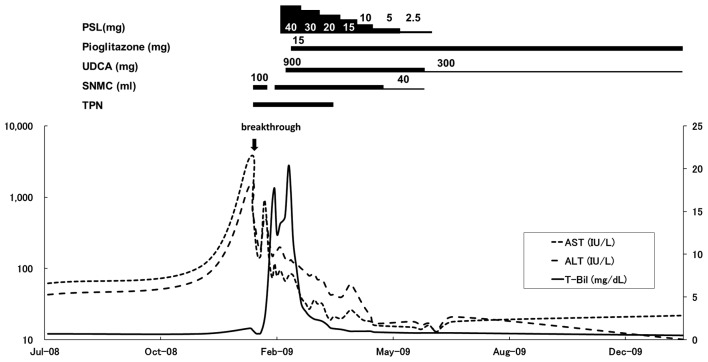
Clinical course. PSL, prednisolone, UDCA, ursodeoxycholic acid; SNMC, Stronger Neo-Minophagen C; TPN, total parenteral nutrition; AST, aspartate aminotransferase; ALT, alanine aminotransferase; T-Bil, total bilirubin.

**Figure 3:**
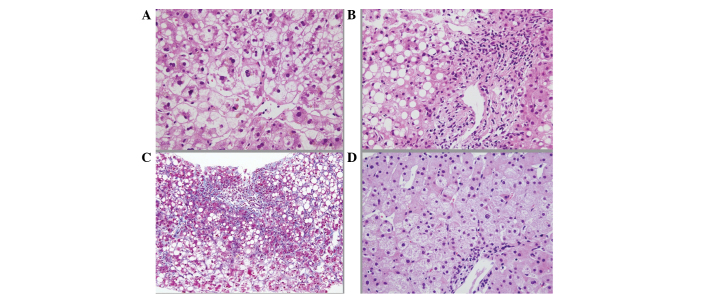
Microscopic findings of the liver biopsy specimen showed (A) steatosis, ballooning degeneration, (B) lobular inflammation, and (C) zone III perisinusoidal fibrosis on admission. (A and B) Hematoxylin and eosin staining (magnification, ×40) and (C) Masson’s trichrome staining (magnification, ×20). (D) All morphological findings were ameliorated after 1 year (hematoxylin and eosin staining; magnification, ×40).

TPN was initiated at 1,000 kcal/day (20 kcal/kg standard body weight/day or 40 kcal/kg actual body weight/day). Phosphorus was also added to the TPN to avoid refeeding syndrome. In addition, 100 ml Stronger Neo-Minophagen C (SNMC; Minophagen Pharmaceutical Co., Ltd., Tokyo, Japan) was administered every day to improve the AST and ALT levels, but was discontinued due to secondary aldosteronism on the eighth day, resulting in increases in AST and ALT levels (Fig. 2). SNMC treatment was resumed on the 13th day, and the serum AST and ALT levels were reduced to 74 and 150 IU/l, respectively. However, total bilirubin levels increased to 15.9 mg/dl on the 20th day. To confirm the diagnosis of liver dysfunction and to rule out liver infection prior to the administration of prednisolone (PSL), percutaneous needle biopsy of the liver was conducted on the 21st day. The pathological findings revealed: i) Macrovesicular steatosis up to 50% of the tissue area; ii) zone 3 perisinusoidal fibrosis with extensive periportal fibrosis; iii) neutrophilic infiltration; iv) mitochondrial swelling; v) wide spread hepatocyte ballooning; and vi) intrabiliary canalicular cholestasis. These histological findings were compatible with NASH (6,12) and thus led to a diagnosis of severe acute exacerbation of secondary NASH with AN (Fig. 3A–C). Thereafter, 40 mg PSL was administered daily. However, the effectiveness of PSL in the treatment of the increasing jaundice was limited. Therefore, 900 mg ursodeoxycholic acid (UDCA) was administered (Fig. 2).

Despite the combined treatment with PSL, SNMC, TPN and UDCA, jaundice continued to increased and the AST and ALT abnormalities were prolonged (Fig. 2). A previous study showed that the prognosis of patients that were similar to this case, who did not respond to TPN refeeding therapy, was extremely poor, mainly due to the late onset of hepatic failure (4). Therefore, once the patient’s family were informed of the risks of pioglitazone for secondary NASH with AN, 15 mg pioglitazone was administered daily starting on the 31st day. The serum AST and ALT levels were reduced gradually thereafter and total bilirubin levels showed a rapid reduction. Liver function was completely normalized on the 105th day (Fig. 2). The liver function remained normal after SNMC and PSL treatment were tapered-off and the UDCA dosage was reduced to 300 mg per day. The patient became able to walk following physical rehabilitation and was discharged on the 168th day.

One year later, nutritional assessment, abdominal CT, abdominal US and liver biopsy tests were performed to evaluate the effectiveness of pioglitazone. Although the total energy intake of the patient was 1,000 kcal/day, which showed no change compared with that prior to treatment, 8 kg of body weight had been gained. In the CT findings, the liver attenuation was 67.0 Hu and the liver/spleen attenuation ratio was 1.11 (Fig. 1c), which were almost the same as the findings on admission. However, the volume of subcutaneous, visceral and trabecular fat tissues had markedly increased. Abdominal US (Fig. 1D) also showed no clear changes compared with that on admission. The histological findings of the liver needle biopsy specimen (Fig. 3D) showed remarkable improvements in the fibrosis, steatosis, inflammation and hepatocyte ballooning.

## Discussion

An increase in the number of obese individuals in the population is of major concern worldwide. NASH is one of the manifestations of obesity and its associated diseases (13). Simultaneously, the number of patients with eating disorders (3) has also increased among young women in developed countries, which is considered to be associated with cultural transition and globalization, including modernization, urbanization and media-exposure promoting the Western beauty-ideal (14). A previous study showed that elevated transaminase levels were observed in ~30% of patients with AN (3) and its histological findings were compatible with secondary NASH (4). Severe weight loss-associated fatty liver disease has also been observed in protein energy malnutrition (15) and postoperative jejunoileal bypass (16).

The mechanisms of acute liver failure in secondary NASH with AN are considered as follows: i) Circulatory disturbance of the liver due to dehydration; and ii) starvation-induced autophagy in the liver (7). Although the outcome of malnutrition-associated NASH is usually favorable with a rapid recover following treatment with TPN (7–9), there have been certain cases with fatal outcomes regardless of TPN and additional intensive therapy, including hemodialysis or glucocorticoid administration (4,17,18). For these patients with secondary NASH a novel therapeutic treatment is urgently required.

Pioglitazone is an oral antidiabetic agent, which improves insulin sensitivity through activating the nuclear peroxisome proliferator activated receptor-γ (PPAR-γ). Clinically, this mechanism of pioglitazone results in improved glycemic control and decreased hepatic fat content. Additional studies have shown that pioglitazone is a promising treatment agent for NASH (11,19). Furthermore, a previous study showed that pioglitazone was also effective for alcoholic steatohepatitis in rats without altering insulin sensitivity (20). Pioglitazone promotes the differentiation of pre-adipocytes into adipocytes (21–23) and may result in the redistribution of triglycerides from the liver into proliferating adipocytes. Pioglitazone is generally known to cause weight gain as an adverse effect, which is supported in this case as the patient gained 8 kg of body weight. From these findings, it appears that pioglitazone improved liver dysfunction simply through weight gain as its adverse effect. Thus, pioglitazone may have direct or indirect effects for patients with secondary NASH with AN without altering insulin sensitivity.

There are limitations to this case report. There may be ethical issues regarding the use of pharmaceuticals of this family in patients with severe liver dysfunction because idiosyncratic hepatotoxic injury has been reported previously for a PPAR-γ agonist (24). However, this was for troglitazone exclusively and, to the best of our knowledge, pioglitazone has never caused such adverse effects. Furthermore, several studies of pioglitazone in the treatment of patients with NASH have confirmed its safety for use in patients with liver dysfunction (11,19). It may be questioned whether pioglitazone was effective, since the serum ALT and AST levels were tending to decrease before the drug treatment was initiated. Even if pioglitazone was effective, it remains unclear whether the effects were direct. In the present case, glucocorticoid, SNMC, TPN and UDCA were also administered. TPN and glucocorticoids are known to promote hepatic steatosis (25,26), while the effects of UDCA have yet to be confirmed on NASH in randomized controlled studies (27). For SNMC, to the best of our knowledge, there are no previous studies determining its effects on NASH. In the present study, the histological improvements of the patient’s liver biopsy specimen and normalization in liver function after a year are not likely to be due to the agents PSL, TPN, UDCA or SNMC, as the administration of these agents had already ended or tapered at the point of histological re-examination. Therefore, it may be suggested that the improvements were the result of pioglitazone treatment. These findings support our hypothesis that pioglitazone had a critical role in both clinical and histological improvements of the patient’s liver.

Notably, although >50% macrovesicular fatty changes were observed in the liver biopsy specimen, the liver-spleen attenuation ratio analyzed from the CT scan was >0.9, which was greater than that defined for fatty liver. Additionally, hepato-renal contrast was not observed in abdominal US. A previous study showed the sensitivity of US for detecting steatosis in patients with nonalcoholic fatty liver disease was 100%, but was reduced to 77.8% in patients with advanced histological fibrosis, while the sensitivity of CT scanning was 69.8 and 48.9% respectively, suggesting that the advanced fibrosis may have interfered with the detection of steatosis by such imaging modalities (28). This report may explain the occult liver findings in the CT and US results in the present case who exhibited moderate fibrosis in the liver.

In conclusion, it is noteworthy that the results of the present case indicated the effectiveness of pioglitazone on secondary NASH with AN in both serological and histological findings. In addition, critical adverse events of pioglitazone were not observed in the present case. Therefore, the administration of pioglitazone for acute exacerbation of secondary NASH with AN may be considered when conventional therapies are not effective as their outcomes are very poor.

## Figures and Tables

**Table I:** Laboratory findings on admission.

Variable	Result
Complete blood count	
WBC	3,100 cells/μl
RBC	374 cells/μl
Hb	12.1 g/dl
Ht	36.4%
PLT	9,6000 cells/μl
Neut	79.3%
Lymph	18.2%
Mono	2.1%
Eos	0.2%
Baso	0.3%
Coagulation	
PT	52%
APTT	28.5 sec
FDP	71.9 mg/dl
Viral markers	
IgM-anti-HA	(−)
HBsAg	(−)
Anti-HCV	(−)
IgG-anti-VCA	×320
IgG-anti-EBNA	×10
IgM-anti-CMV	(−)
Serological tests	
IgA	262 mg/dl
IgM	63 mg/dl
IgG	946 mg/dl
Autoantibody	
Anti-microsome	(−)
Anti-tyroglobulin	(−)
ANA	640 fold
AMA(M2)	(−)
Blood chemistry	
AST	3,665 IU/l
ALT	1,495 IU/l
ALP	1,152 IU/l
CHE	157 IU/l
γ-GTP	237 IU/l
LDH	1,594 IU/l
T.Bil	1.35 mg/dl
D-Bil	0.41 mg/dl
Alb	3.8 g/dl
BUN	94.5 mg/dl
Cr	1.67 mg/dl
UA	8.1 mg/dl
CPK	265 IU/l
LDL	66 mg/dl
TG	23 mg/dl
HDL	137 mg/dl
Amy	288 IU/l
Na	145 mEq/l
K	6.52 mEq/l
Cl	112 mEq/l
NH_3_	35 μg/dl
CRP	0 mg/dl
RF	11.4 IU/ml
FBS	52 mg/dl
Hormone	
Free T3	<1.0 pg/ml
Free T4	1.79 ng/dl
TSH	1.85 μU/ml
IRI	0.46 μIU/ml
IRG	110 pg/ml
ACTH	53.6 pg/ml
Cortisol	26.3 μg/dl
Others	
β-D-G	6.6 pg/ml

WBC, white blood cells; RBC, red blood cells; Hb, hemoglobin; Ht, hematocrit; PLT, platelet count; Neut, neutrophils; Lymp, lymphocytes; Mono, monophils; Eos, eosinophils; Baso, basophils; PT, prothrombin time; APTT, activated partial thromboplastin time; FDP, fibrin degradation products; IgM, immunoglobulin M; HA, hemagglutinin; HBsAg, hepatitis B surface antigen; HCV, hepatitis C virus; IgG, immunoglobulin G; VCA, viral capsid antigen; EBNA, Epstein Barr nuclear antigen; CMV, cytomegalovirus; IgA, immunoglobulin A; ANA, antinuclear antibody; AMA, antimitochondrial antibody; AST, aspartate aminotransferase; ALT, alanine aminotransferase; ALP, alkaline phosphatase; CHE, cholinesterase; γ-GTP, γ-glutamyl transpeptidase; LDH, lactose dehydrogenase; T.Bil, total bilirubin; D-Bil, direct bilirubin; Alb, albumin; BUN, blood urea nitrogen; Cr, creatinine; UA, uric acid; CPK, creatinine phosphokinase; LDL, low density lipoproteins; TG, triglycerides; HDL, high density lipoproteins; Amy, amylase; CRP, C-reactive protein; RF, rheumatoid factor; FBS, fasting blood sugar; TSH, thyroid-stimulating hormone; IRI, immunoreactive insulin; IRG, immunoreactive glucagon; ACTH, adrenocorticotropin; β-D-G, β-D-glucose.

## Abbreviations

ALT: alanine aminotransferase
AN: anorexia nervosa
AST: aspartate aminotransferase
ASH: alcoholic steatohepatitis
BMI: body mass index
CT: computed tomography
NASH: nonalcoholic steatohepatitis
PPARγ: peroxisome proliferator activated receptor-γ
PSL: prednisolone
PT: prothrombin time
SNMC: Stronger Neo-Minophagen C
TPN: total parenteral nutrition
UDCA: ursodeoxycholic acid
US: ultrasonography
